# New Psychoactive Substances Toxicity: A Systematic Review of Acute and Chronic Psychiatric Effects

**DOI:** 10.3390/ijms25179484

**Published:** 2024-08-31

**Authors:** Beldisa Taflaj, Nunzia La Maida, Roberta Tittarelli, Annagiulia Di Trana, Ilaria D’Acquarica

**Affiliations:** 1 Azienda Ospedaliera San Giovanni Addolorata, Via dell’Amba Aradam 8, 00184 Rome, Italy; btaflaj@hsangiovanni.roma.it; 2 National Centre on Addiction and Doping, Istituto Superiore di Sanità, Viale Regina Elena 299, 00161 Rome, Italy; nunzia.lamaida@iss.it; 3 Laboratory of Forensic Toxicology, Section of Legal and Forensic Medicine, Social Security and Forensic Toxicology, Department of Biomedicine and Prevention, Faculty of Medicine and Surgery, University of Rome “Tor Vergata”, Via Montpellier 1, 00133 Rome, Italy; roberta.tittarelli@uniroma2.it; 4 Dipartimento di Chimica e Tecnologie del Farmaco, Sapienza Università di Roma, Piazzale A. Moro 5, 00185 Rome, Italy; ilaria.dacquarica@uniroma1.it

**Keywords:** new psychoactive substances, psychotic disorders, psychiatric symptoms, mental disorders, substance-related disorders, hallucinations, schizophrenia, bipolar disorder

## Abstract

New psychoactive substances (NPSs) are a heterogenous group of psychotropic molecules and diverted pharmaceutical drugs sold worldwide as legal substitutes for controlled drugs. The psychiatric consequences of NPS use are relatively unknown, although evidence of related psychotic symptoms has been described in the literature. We sought to summarize the available evidence on NPS-related psychiatric disorders, to facilitate the interpretation of the molecular mechanism underlying their specific pathologies. A literature search of Scopus, PubMed and Google Scholar was conducted including studies published between 2013 and 2024, in which a correlation between NPS consumption and psychiatric symptoms was reported. Furthermore, the short- and long-term psychopathological effects were included. The literature search resulted in 109 NPS-related intoxication cases in which acute or chronic psychiatric symptoms were reported, mostly related to synthetic cannabinoids, followed by synthetic cathinones, hallucinogens, natural NPSs and stimulants. The most common acute symptoms were hallucinations, aggressiveness, and psychotic and bizarre behavior, related to the molecular disbalance of neurotransmitters in the central nervous systems, with different mechanisms. The lack of clear diagnostic criteria and toxicological analyses has resulted in crucial complications in psychiatric diagnoses related to NPS intoxication. Hence, the implementation of toxicological screening procedures in emergency rooms, including the main NPS classes, should support the diagnosis of acute intoxication and its proper therapeutic treatment. Finally, proper follow-up should be implemented to assess the chronic sequelae.

## 1. Introduction

New psychoactive substances (NPS) are a heterogenous group of psychotropic molecules, sold worldwide as legal substitutes for controlled drugs. Moreover, diverted pharmaceutical drugs or unusual consumption settings of banned drugs of abuse are internationally monitored as NPSs, even though some substances are specifically outlawed in several countries, as in the case of ketamine and psilocybin. The rapid spread of NPSs is strongly related to their potency, relatively low production costs and ease of commercialization due to their ambiguous legal status [1,2]. Indeed, their distribution follows unusual preferred trade routes, such as via the Internet, where they are purchased under different names as products “not for human consumption” [1]. Although more than 1200 NPSs are currently known, a total number of 566 NPSs were reported to the United Nations Office on Drugs and Crimes (UNODC) in 2022, belonging to different categories: (1) sedative/hypnotics (e.g., benzodiazepine-type NPSs), (2) dissociatives (e.g., deschloroketamine), (3) hallucinogens (e.g., phenylalkylamine-based and lysergamide-based molecules), (4) stimulants (e.g., phenethylamines, piperazines, synthetic cathinones, SCs and tryptamines), (5) synthetic cannabinoid receptor agonists (SCRAs) and (6) synthetic opioids (e.g., fentanyl analogs, nitazenes) [1,3]. At the end of 2023, the European illegal market was populated by about 400 NPSs, 26 of which were first reported in Europe, mostly SCRAs [4]. The constant evolution of the NPS market represents the most challenging feature of this phenomenon for health personnel, due to the unknown risks related to new substances when they are dropped into the illicit market for the first time [5].

Among the numerous adverse effects related to NPS intoxication, such as hepatotoxicity, respiratory depression, cardiotoxicity and fertility issues, psychiatric effects have recently been raising concerns [6,7]. Depending on their pharmacological classification, NPSs have highly variable mechanisms of action, some of which can potentially induce the onset of psychiatric conditions, producing a wide range of mental state changes, as well as transient psychotic states or even long-lasting psychiatric disorders [8,9]. Furthermore, subjects with psychiatric conditions, either diagnosed or not, appear more prone to NPS consumption than the general population, increasing the risk of severe mental illness or psychiatric disorder exacerbation [9,10]. According to the literature, NPS consumption may exert different acute psychiatric and neurological symptoms, such as anxiety, panic attacks, psychosis, agitated/excited delirium, paranoia and hallucinations confusion, agitation and aggression [8,11,12]. Recently, Schifano et al. investigated the mental health risk posed to the general population by NPS intoxication, focusing on the long-term effects due to NPSs’ interference with the central nervous system dopamine pathway. As a result, various complications were described in this study, such as psychosis [12]. Furthermore, psychotic events were often observed as a result of SCRA consumption by non-psychotic patients [13], or SC consumption [14,15,16]. In 2015, the psychopathological effects caused by different NPS classes were observed by Schifano and colleagues, who described the short- and long-term clinical effects of different NPSs [12].

Notably, NPS consumption often occurs as polydrug abuse, characterized by the concurrent consumption of different classes of NPSs, classic drugs of abuse and diverted pharmaceutical drugs, but also psychotropic prescription drugs such as antipsychotics or hypnotics, further complicating the psychiatric sequelae [17]. Especially in specific settings such as prisons, the concurrent misuse of such substances heavily impacts the conditions and rehabilitation of the subjects, further increasing the physical and mental health risks [18]. Therefore, the lack of analytical evidence of the consumed substance further complicates the elucidation of NPSs’ psychiatric toxicity due to the lack of important information [19].

In this scenario, we sought to systematically review the literature to identify and summarize the available evidence on the relationship between psychiatric disorders and NPS intake, to clarify the connection between NPS use and the development of specific psychopathology.

## 2. Materials and Methods

A comprehensive literature search was performed by the authors to find all the relevant scientific articles reporting cases of NPS abuse in which acute and chronic psychiatric symptoms and/or disorders were sufficiently reported.

Three different electronic databases, Scopus, PubMed and Google Scholar, were searched, considering all the items from 1 January 2013 to 6 April 2024. 

The search terms consisted of the main NPS classes/substances and the psychological/psychopathological outcomes of their abuse. In particular, the following keywords, alone or in combination, were used: new psychoactive substances, designer drugs, novel psychoactive substances, synthetic cathinones, synthetic cannabinoids (SCRAs), synthetic opioids, benzimidazole opioids, fentanyl analogs, tryptamines, hallucinogens, plant-based NPS, natural NPS, phenethylamines, phencyclidines, piperazines, designer benzodiazepines.

Furthermore, Psychotic Disorders OR Psychiatric Symptoms OR Mental Disorders OR Substance-Related Disorders OR Substance Abuse OR Substance Use Disorders OR Psychotic Symptoms OR Hallucinations OR Delusions were the terms used in the query related to psychological/psychopathological outcomes, while we added Schizophrenia OR Bipolar Disorder OR Major Depressive Disorder OR Anxiety Disorders OR Post-Traumatic Stress Disorder descriptors for the major psychiatric diagnosis/outcomes.

Potentially relevant studies were retrieved manually from the reference lists of the screened/selected manuscripts.

**Figure 1:**
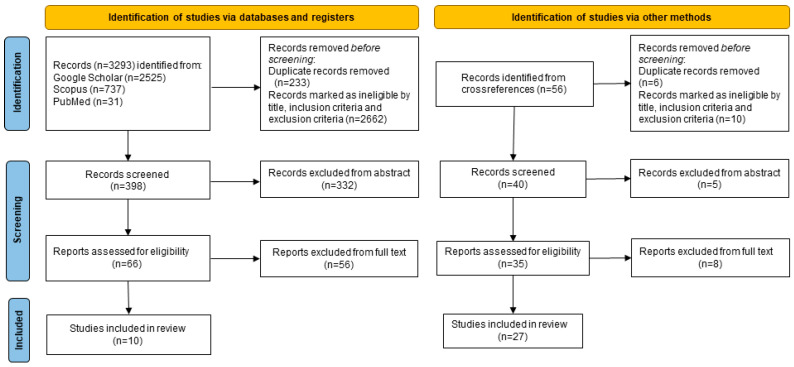
Prisma 2020 flow diagram of the search sources and strategy.

The preferred reporting items for systematic reviews and meta-analysis (PRISMA) statement was the methodology selected for the present review [20]. According to the guidelines of the 2020 PRISMA statement [20], the research team evaluated the following items: the definition of the research question, hypothesis and objectives; a bibliographic search; data collection; screening of the scientific papers selected; and finally, an analysis of the main findings and conclusions, including the strengths and weaknesses of these studies (Figure 1).

The eligibility criteria included only studies describing a correlation between the use of NPSs and psychiatric/psychopathological outcomes, studies with sufficient details on case history, and cases in which an NPS was detected by toxicological analyses or referred to by the patient or their family. Furthermore, studies in English, Italian, French and Spanish were taken into consideration. Case series with large populations with no NPS specified, narrative reviews, book chapters, books, symposiums, letters to the editor and studies not relevant to the topic were excluded.

One of the authors screened all titles and abstracts, assessing 3293 candidates according to the screening criteria. Among these, 233 duplicate records were removed, while 2662 articles were excluded based on title/inclusion/exclusion criteria, and 332 were excluded based on the abstract. Then, the full texts of the remaining articles (n = 66) were independently reviewed by the authors, who determined whether each study met the inclusion criteria. Fifty-six cross-references were identified from each included study, and of these, 6 duplicate items and 10 records marked as ineligible were excluded. The screened records (n = 40) were analyzed and 5 articles were excluded based on the abstract, and 8 based on the full text. There were 27 studies included from the cross-references. A detailed workflow of the review process is provided in the PRISMA flow diagram (Figure 1). There was no restriction on the setting (e.g., inpatient, forensic settings, high schools).

All the authors critically reviewed every study, and the relevant data are given in Table 1. This systematic review protocol was registered in PROSPERO (International Prospective Register of Systematic Review with registration number CRD42024583623, available from 6 September 2024).

## 3. Results

**Figure 2:**
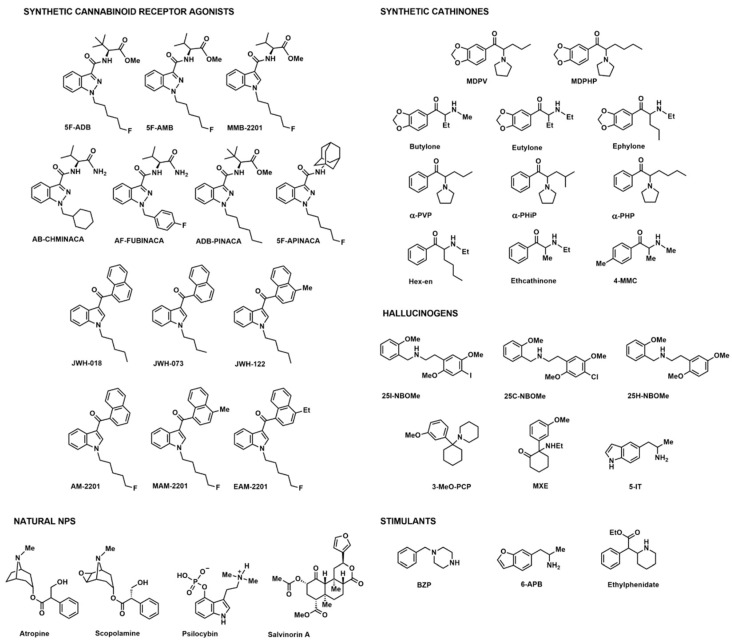
Chemical structures of NPSs involved in 109 related intoxications with acute or chronic psychiatric symptoms, categorized into classes. Abbreviations: MDPV, 3,4-methylenedioxypyrovalerone; MDPHP, 3,4-methylenedioxy-alpha-pyrrolidinohexanophenone; α-PVP, alpha-pyrrolidinovalerophenone; α-PHiP, α-pyrrolidinoisohexanophenone; α-PHP, α-pyrrolidinohexanophenone; Hex-en, N-ethyl-hexedrone; 4-MMC, mephedrone; 3-MeO-PCP, 1-[1-(3-methoxyphenyl)cyclohexyl]piperidine; MXE, methoxetamine; 5-IT, 1-(1H-indol-5-yl)propan-2-amine; BZP, 1-benzylpiperazine; 6-APB, 6-(2-Aminopropyl)benzofuran.

The literature search resulted in 109 NPS-related intoxication cases in which acute or chronic psychiatric symptoms were reported (Table 1). However, a proper diagnosis of psychiatric disorder was provided in only a few cases, reporting Hallucinogen Persisting Perception Disorder (HPPD) [21] or substance-induced psychiatric disorders [22]. A total number of 36 different molecules (Figure 2) were analytically confirmed, although 37 cases reported the generic NPS class.

SCRA-related intoxications were the most reported (n = 57) in subjects with a mean age of 24.4 years (range: 14–59), involving the highest number of pediatric subjects [21,23,24,25,26,27,28,29,30,31,32,33,34,35,36,37,38]. SCRAs were consumed either alone or in combination (n = 19) with other drugs, mostly illegal cannabinoids and NPSs [25,31,32,33,34,35,37,38]. Unfortunately, the declared molecules or the mixtures had not been verified by toxicological analysis in more than 50% of cases, correlating the psychiatric symptoms to the general class of NPS or reporting only the NPS’s commercial name [21,32,33,34,35,36,37,38]. Concerning SCRA-related cases, 6 molecules were analytically confirmed in 25 different intoxications, with AM-2201 being the most recurrent (n = 8) [24,27,32]. While acute symptoms were reported in all the cases, chronic sequelae of SCRA intoxication were reported in almost 8% of cases. The acute psychiatric symptoms were mostly hallucinations (n = 51) [21,23,24,28,31,32,33,38], aggressiveness (n = 24) [24,26,32,33,34,38], anxiety (n = 14) [23,24,28,33,35], paranoia (n = 11) [32,34] and psychosis (n = 11) [32,34]. Furthermore, subjects showed increased sexuality and disinhibited behavior, suggesting an empathogenic-like effect [38]. Interestingly, polydrug consumption with SCRAs increased suicidality and enhanced THC effects, with chronic episodes of hallucination and psychosis.

SCs were reported in 29 different cases, including older subjects than other NPS class users (mean age of 30.9 years, range: 17–40 years old) [16,19,22,39,40,41,42,43,44,45,46,47,48,49,50]. Similarly to SCRAs, the consumed drug was not analytically confirmed, but the medical personnel based the diagnosis on the patient declaration [39,48]. However, mephedrone (4-MMC) was the most reported among the SCs (28% cases) [16,22,42,43], followed by methylenedioxypyrovalerone (MDPV, 21% cases) [16,19,40,41,49]. Interestingly, MDPV was prevalently consumed in combination with other drugs, while 4-MMC was indifferently consumed alone or in combination (50% vs. 50%). One-third of cases involved subjects with familial or personal psychiatric history, mostly bipolar disorder [16,19,41,45], or a substance use disorder related to classic drugs of abuse and NPSs [22,39,41,42,43,45,48]. The most frequent acute symptom was hallucination (n = 7) [16,19,39,40,41,42,43,46,47,48,49], followed by psychosis, paranoia and anxiety. Suicidal behavior was prevalently reported as related to MDPV consumption [16,49], while depressive mood was related to N-ethylcathinone [16].

Among hallucinogenic NPS intoxications, phenethylamines (n = 16) and phencyclidines (n = 1) were reported in a total of 17 cases. Subjects had a mean age of 25 years old (range: 17–53). The most representative hallucinogen was phenethylamine 25I-NBOMe (n = 9) [19,51,52], followed by 5-IT (n = 6) [53], while the only reported phencyclidine, 3-MeO-PCP, involved a subject with anxious–depressive symptoms [54]. In one case, a mix of NBOMe molecules was consumed [19], while association with other psychotropic substances was reported in 30% of cases [19,51]. Besides the expected hallucination, other psychiatric symptoms reported were aggressiveness and agitation (70% cases) [52,53]. Concerning the psychiatric history of subjects, schizophrenia was reported in one case [51]. Notably, two cases lacked acute symptoms, while mid-term symptoms were listed, like persistent memory impairment [19]. Generic psychiatric symptoms such as restlessness and pathological agitation were reported in 5-IT intoxications [53]. Otherwise, the methoxetamine-related acute symptoms were dissociation, hallucination and motivational anhedonia, resulting in a diagnosis of substance-induced psychosis after 3 months [55].

Only three cases concerned natural NPSs of different pharmacological classes, in particular, two hallucinogens and one stimulant [19,21]. Two out of three cases involved subjects with a previous diagnosis of schizophrenia (n = 1) or bipolar I disorder (n = 1) associated with regular substance abuse [19]. The psychiatric condition worsened in both cases after NPS consumption. Similarly, stimulant NPSs were reported in only three cases, in which subjects with diagnosed schizophrenia showed increased aggressiveness and bizarre behavior [19,24]. 

**Table 1:** Acute and/or chronic psychiatric symptoms in NPS-related intoxications reported in the literature between 1 January 2013 and 6 April 2024.

New Psychoactive Substance	Age, Sex Clinical History	Psychiatric Comorbidities	Other Drugs	Psychiatric Symptoms	Year	Ref.
SCRAs = 57						
5F-ADB	17 yo, male	na	nr	Psychomotor agitation, confusion, anxiety, psychosis, delirium/hallucinations,	2017	[23]
5F-ADB	17 yo, male	na	nr	Psychomotor agitation, confusion, anxiety psychosis, delirium/hallucination	2017	[23]
5F-ADB	26 yo, male	na	nr	Bizarre behavior, changing moods, incessant oral fluency, nonsensical statements	2019	[25]
5F-ADB	49 yo, male	na	Opioids, fentanyl	Substance-induced psychosis, suicidal behavior	2019	[25]
5F-ADB	31 yo, male	na	nr	Panic attacks	2023	[24]
5F-ADB MMB-2201 “Cherry bomb formula 6A”	14 yo, female	na	THC	Altered consciousness, c, apparent seizures	2017	[23]
5F-ADBSpice	21 yo, male	na	THC	Psychomotor agitation, suicidality, altered language, bradypsychia	2017	[23]
JWH-122	21 yo, male	na	THC, 6-APB	Agitation, paranoid behavior	2018	[32]
AB-CHMINACA AB-FUBINACA AM-2201 5F-AMB 5F-APINACA EAM-2201 JWH-018 JWH-122 MAM-2201	25 yo, male	na	na	Psychosis, anxiety, panic attacks, agitation	2023	[24]
ADB-PINACA	25 yo, male	na	nr	Restless and aggressive behavior	2015	[26]
ADB-PINACA	24 yo, male	na	nr	Confusion and agitation	2015	[26]
ADB-PINACA	30 yo, male regular cocaine user	na	nr	Severely combative and aggressive behavior	2015	[26]
ADB-PINACA	16 yo, female	na	nr	Mild agitation and anxiety	2015	[26]
AM-2201	26 yo, male regular THC user	na	nr	Severe panic attacks, recurring visual disturbances, impairment in social and occupational functioning	2015	[27]
AM-2201	35 yo, male	paranoid schizophrenia diagnosis, antisocial personality disorder	nr	Manic, prominent behavioral changes	2014	[28]
AM-2201	21 yo, male	paranoid schizophrenia	nr	Severe agitation, anxiety and presence of paranoid delusions	2014	[28]
AM-2201	27 yo, male	undifferentiated schizophrenia	nr	Hypomanic severe anxiety and agitation, behavioral changes, possible haptic hallucinations	2014	[28]
AM-2201	29 yo, male	undifferentiated schizophrenia	nr	Agitation, severe formal thought symptoms, moderate anxiety	2014	[28]
AM-2201	18 yo, male	na	na	Aggressive behavior	2023	[24]
AM-2201 JWH-073 JWH-018	23 yo, male	na	nr	Psychotic episodes	2013	[29]
JWH-018	17 yo, female	na	Cannabis	Violent behavior, hallucination	2018	[32]
JWH-018 JWH-122	17 yo, male	na	nr	Altered state of mind	2015	[30]
JWH-122	18 yo, male	na	THC	Acute: hallucinations Chronic: occasional visual disorders hallucinations, derealization and body lightness	2017	[31]
JWH-122	18 yo, male	na	na	Hallucinations, perception disorder	2023	[24]
K2	19 yo, male	na	THC	Combativeness	2013	[33]
K2	24 yo, male	na	nr	Anxiety	2013	[33]
K2	22 yo, male	na	nr	Hallucinations, agitation, dream state	2013	[33]
K2	17 yo, male	na	nr	Hallucinations, catatonia, disorganized thoughts	2018	[32]
K2	36 yo, male	na	Ephedrine, pseudoephedrine, promethazine, DXM	Florid persecutory delusions, hallucinations	2018	[32]
K2	36 yo, male THC addiction	schizophrenia	THC	Worsening paranoia, illogical speech, hallucinations	2018	[32]
K2 Spice	18 yo, male	na	THC	Delusions, disorganized behavior	2018	[32]
K2 (Bayou Blaster)	19 yo, female	na	nr	Agitation, altered mental status, drowsiness, depression, suicidal ideations	2013	[33]
SCRAs (Mr. Nice Guy)	23 yo, male	na	Cannabinoids	Psychotic symptoms, persecutory delusions, agitation, aggression, paranoia, altered mental status and severe agitation	2015	[34]
SCRAs (Humboldt Gold)	17 yo, male	na	Cannabinoids	Agitation, hallucinations	2013	[33]
SCRAs (Space)	17 yo, male	na	nr	Self-perception disorder, anxiety	2013	[33]
SCRAs	18 yo, male	na	BDZs	Anxiety, insomnia, ideas of reference hallucinations	2013	[35]
SCRAs	20 yo, male regular THC user	na	nr	Acute: disturbance in consciousness, change in cognition, delirium, psychomotor agitation, thought-blocking, disorganized behaviors and thoughts Chronic: diagnosed with schizophreniform disorder	2013	[36]
SCRAs	18 yo, male	na	Illicit stimulants, cannabinoids	Acute: mystical and grandiose delusions, substance-induced bipolar disorder Chronic: mystical and grandiose delusions	2015	[37]
SCRAs	28 yo, male	paranoid schizophrenia	nr	Delusional mood, persecutory delusions, hallucinations, disorganized thoughts	2018	[38]
SCRAs	32 yo, female regular SCRA, THC, crack cocaine and heroin user	schizoaffective disorder	Aripiprazole, carbolithium	Aggressive behavior, sexual disinhibition, delusional mood, grandiose and persecutory delusions	2018	[38]
SCRAs	20 yo, male	na	Polysubstance misuse	Bizarre behavior, substance-induced psychotic episodes, sexual disinhibition, arousal and aggressive behavior	2018	[38]
SCRAs	39 yo, male regular polysubstance user	bipolar disorder	nr	Agitation, aggressive behavior, disordered with grandiose delusions	2018	[38]
SCRAs	31 yo, male occasional LSD user; ecstasy, cannabinoids and psychostimulants	na	nr	Acute: HPPD Type II Chronic: perception disorders	2018	[21]
SCRAs	18 yo, male	na	nr	Self-talking and laughing, delusions, manic symptoms	2018	[32]
SCRAs	18 yo, female occasional THC user	na	nr	Hallucinations, soliloquy	2018	[32]
SCRAs	17 yo, male regular THC, LSD and ecstasy user	na	nr	Paranoia and disorganized thought, bizarre behavior	2018	[32]
SCRAs (Black Diamond)	26 yo, male	na	nr	Paranoid delusions	2018	[32]
SCRAs (bonsai)	17 yo, male	na	nr	Capgras syndrome, persecutory delusions, hallucination	2018	[32]
SCRAs (bonsai)	31 yo, male	na	nr	Anger, insomnia, delusions	2018	[32]
SCRAs (kush)	21 yo, male	na	THC	Catatonia, self-talk, inappropriate laughter	2018	[32]
SCRAs (Mr. Nice Guy)	23 yo, male	na	nr	Visibly psychotic, persecutory delusions	2018	[32]
SCRAs (spice)	17 yo, male regular THC user	na	nr	Catatonia, delusions	2018	[32]
SCRAs (spice)	17 yo, male	na	LSD, psilocybin, SCs, oxycodone	Delusions, hallucinations	2018	[32]
Spice	59 yo, male previous regular heroin, cocaine and THC user	PTSD	nr	Hallucinations, disorganized, bizarre behavior	2018	[32]
Spice	20 yo, male	na	nr	Paranoia, hallucinations	2018	[32]
Spice	23 yo, male	na	Cannabis	Nonsensical speech, paranoia	2018	[32]
Spice	25 yo, male	na	nr	Severe psychosis, paranoia	2018	[32]
SCs (n = 29)						
Bath salts	26 yo, female regular SC user	na	na	After 5 months: visual hallucinations After 8 months: occasional hallucinations, reduced psychotic symptoms	2013	[39]
Bath salts	40 yo, male regular THC user	na	nr	Paranoid behavior, hallucinations, psychological delusions, psychosis, aggressive behavior, self-mutilation	2017	[48]
Bath salts	29 yo, female	bipolar disorder	Polysubstance abuse	Altered mental state	2016	[19]
Bath salts	23 yo, male	na	nr	Bizarre behavior, suicidality, hallucinations	2016	[19]
MDPV 4-MMC	18 yo, male	na	Cannabinoids	Acute: hallucinations, agitation, confusion, memory loss After 1 week: memory and speech disorder	2014	[40]
MDPV Butylone	28 yo, male	na	na	Acute psychosis	2016	[19]
Eutylone	32 yo, male	bipolar affective disorder schizophrenia	Ethanol	Abnormal behavior, unconsciousness	2023	[16]
MDPHP	nr, male	na	Ethanol, BDZs	Aggressive behavior	2023	[16]
MDPV	23 yo, male	psychiatric history	THC	Bizarre behavior, suicidality, hallucinations, agitation	2023	[16]
MDPV	40 yo, male	bipolar disorder history	Lidocaine	Aggressive behavior, delusional	2023	[16]
MDPV 4-MMC	33 yo, male opiates and MAMP addiction	familial bipolar disorder	nr	Hallucinations (auditory and visual), anxiety, paranoia, withdrawal syndrome	2013	[41]
MDPV 4-MMC butylone α-PVP	46 yo, male	na	Zolpidem	Suicidal behavior hallucinations, paranoia, anxiety persecutory delusions	2014	[49]
4-MMC	40 yo, male regular cocaine user	na	nr	Acute: persecution and reference delusions Chronic: delusions	2016	[42]
4-MMC	26 yo, male heavy ethanol userand regular cocaine user	na	nr	Hallucinations	2016	[42]
4-MMC	25 yo, male regular cocaine, KETA, GHB, MDMA, MAMP and poppers user	antisocial behavior	nr	Paranoid behavior, intense emotional and behavioral impact, hallucinations	2016	[43]
4-MMC	36 yo, male	na	Cocaine MDMA BDZs	Aggressive and bizarre behavior	2023	[16]
4-MMC α-PVP	26 yo, male occasional ethanol, cannabinoid, AMP and 4-MMC user	na	nr	Acute: psychotic disorder Chronic: craving, withdrawal syndrome, addiction syndrome, drug tolerance, substance-induced schizophrenia-like psychosis diagnosis	2023	[22]
Ethcathinone	nr, male	na	AMP	Depressed mood	2023	[16]
Hex-en	21 yo, male	na	BDZs, AMP, cannabinoids	Disorientation, aggressive behavior	2023	[16]
Ephylone	32 yo, male	na	nr	Psychomotor agitation, aggressive behavior	2019	[44]
Ephylone	26 yo, female	na	MDMA	Disconnected speech, episodes of visual hallucinations,	2019	[44]
Ephylone	26 yo, male	history of mental disorders	nr	Psychosis, paranoia, inconsistent speech	2019	[44]
Ephylone	18 yo, male	na	nr	Psychomotor agitation	2019	[44]
Ephylone	29 yo, male regular MDMA and SCs user	history of bipolar disorder	AMP, BDZs, cannabinoids, opiates	Agitation, aggressive behavior	2017	[45]
SCs	45 yo, male	paranoid schizophrenia mood disorder	nr	Chronic: increasing agitation	2016	[19]
α-PHiP	37 yo, male	na	na	Agitation and bizarre behavior	2023	[50]
α-PHP	39 yo, male	na	nr	Hallucinations, delusions, aggressive behavior, anxiety, psychotic symptoms	2018	[46]
α-PVP	17 yo, female	na	nr	Altered mental status, agitation, psychotic behaviors, auditory hallucinations	2016	[47]
α-PVP	40 yo, male	na	na	Psychotic behavior	2023	[50]
Hallucinogens (n = 17)						
25I-NBOMe	29 yo, male	na	AMP MDMA, 2C-I	Agitation, aggressiveness, self-injury	2013	[51]
25I-NBOMe	20 yo, male	na	2C-I	Agitation, visual hallucinations	2013	[51]
25I-NBOMe	19 yo, male	na	2C-I	Agitation, auditory and visual hallucinations	2013	[51]
25I-NBOMe	22 yo, male	na	2C-I	Agitation, visual hallucinations, aggressiveness	2013	[51]
25I-NBOMe	21 yo, male history of 2C-B use	na	2C-I	Agitation, visual hallucinations, aggressiveness	2013	[51]
25I-NBOMe	20 yo, male regular AMP and MDMA user	na	Ethanol, 2C-I	Visual hallucinations	2013	[51]
25I-NBOMe	20 yo male regular MDMA, cocaine, cannabis and LSD user	na	2C-I	Visual hallucinations	2013	[51]
25I-NBOMe	18 yo, male	na	Cannabinoids (screening)	Acute: severe agitation, hallucinations Chronic: episodes of aggressiveness	2013	[52]
25I-NBOMe 25C-NBOMe 25H-NBOMe	29 yo, nr, regular THC and LSD user	schizophrenia	nr	After 1 month: persistent memory impairment, significant abnormalities in executive functions	2016	[19]
3-MeO-PCP	29 yo, male	anxious–depressive symptoms in adolescence under pharmacological treatment	nr	After 3 days: mania-like episode with psychotic features, visual and tactile hallucinations, paranoid delusions, severe dissociation, sense of impending doom, psychomotor agitation, aggressive behaviors, persistent psychotic symptoms and behavioral alterations, substance-induced psychosis	2024	[54]
5-IT	24 yo, male	na	nr	Hallucinations, restlessness	2014	[53]
5-IT	53 yo, male	na	MXE, ethanol	Restlessness	2014	[53]
5-IT	21 yo, male	na	Methylphenidate, ritalinic acid, KETA	Agitation, restlessness	2014	[53]
5-IT	27 yo, female	na	MDPV, ethylphenidate, 4-MEC, 5-APB, buprenorphine, ethanol	Hallucinations, restlessness	2014	[53]
5-IT	23 yo, male	na	na	Hallucinations, agitation, restlessness	2014	[53]
5-IT	23 yo, male	na	na	Agitation	2014	[53]
MXE	23 yo, male occasional THC, KETA and LSD user	na	nr	Acute: usual visual and auditory hallucinosis, severe dissociative symptoms, detachment from reality and absorption in imaginative thoughts, marked affective withdrawal, motivational anhedonia After 3 months: diagnosis of substance-induced psychotic disorder	2019	[55]
Natural NPS (n = 3)						
*Datura stramonium* (tropane alkaloids)	32 yo, male regular hallucinogen user	paranoid schizophrenia with mental and behavioral disorders	na	Paranoid schizophrenia, mental and behavioral disorders	2016	[19]
*Psilocybe* spp. (psilocybin)	23 yo, male regular THC user	na	Ethanol, THC	Acute: hallucinations Chronic: HPPD Type I	2018	[21]
*Salvia divinorum* (salvinorin A)	24 yo, female chronic salvinorin A user	bipolar I disorder, occasional psychotic symptoms	nr	Psychotic symptoms, auditory hallucinations, persecutory and religious delusions	2016	[19]
Stimulants (n = 3)						
BZP	48 yo, male	schizophrenia	nr	Aggressive behavior, incoherent speech	2016	[19]
6-APB	21 yo, male	na	THC	Acute psychosis, agitation, paranoid behavior	2023	[24]
Ethylphenidate	30 yo, male	paranoid schizophrenia	Benzocaine	Severe thought disorder, chaotic and bizarre behavior pattern	2016	[19]

Abbreviations: yo, years-old; nr, not reported; na, not available; 5F-ADB, (*S*)-methyl 2-[[1-(5-fluoropentyl)indazole-3-carbonyl]amino]-3,3-dimethylbutanoate; MMB-2201, (*S*)-methyl 2-[[1-(5-fluoropentyl)indole-3-carbonyl]amino]-3-methylbutanoate; THC, Δ9-tetrahydrocannabinol; 6-APB, 6-(2-Aminopropyl)benzofuran; JWH-122, (4-methyl-1-naphthyl)-(1-pentylindol-3-yl)methanone; AB-CHMINACA, N-(1-amino-3-methyl-1-oxobutan-2-yl)-1-(cyclohexylmethyl)-1H-indazole-3-carboxamide; AB-FUBINACA, N-(1-amino-3-methyl-1-oxobutan-2-yl)-1-(4-fluorobenzyl)-1H-indazole-3-carboxamide; AM-2201, [1-(5-fluoropentyl)-1H-indol-3-yl](naphthalen-1-yl)methanone; 5F-AMB, (*S*)-methyl 2-({[1-(5-fluoropentyl)-1H-indazol-3-yl]carbonyl}amino)-3-methylbutanoate; 5F-APINACA, N-(1-adamantyl)-1-(5-fluoropentyl)indazole-3-carboxamide; EAM-2201, [1-(5-fluoropentyl)-1H-indol-3-yl]-(4-ethyl-naphthalen-1-yl)methanone; JWH-018, naphthalen-1-yl(1-pentyl-1H-indol-3-yl)methanone; MAM-2201, 1-(5-fluoropentyl)-3-(4-methyl-naphthoyl)indole; ADB-pinaca, N-(1-amino-3,3-dimethyl-1-oxobutan-2-yl)-1-pentyl-1H-indazole-3-carboxamide; JWH-073, (1-butyl-1H-indol-3-yl)(naphthalen-1-yl)methanone; DXM, dextromethorphan; LSD, d-Lysergic Acid Diethylamide; SCRAs, synthetic cannabinoid receptor agonists; PTSD, Post-Traumatic Stress Disorder; HPPD, Hallucinogen Persisting Perception Disorder; BDZs, benzodiazepines; MDPV, 3,4-methylenedioxypyrovalerone; 4-MMC, mephedrone; MAMP, methamphetamine; KETA, ketamine; GHB, γ-hydroxybutyric acid; MDMA, methylendioxymethamphetamine; MDPHP, 3,4-methylenedioxy-alpha-pyrrolidinohexanophenone; α-PVP, alpha-pyrrolidinovalerophenone; AMP, amphetamine; Hex-en, N-ethyl-hexedrone; SCs, synthetic cathinones; α-PHiP, α-pyrrolidinoisohexanophenone; α-PHP, α-pyrrolidinohexanophenone; 25I-NBOMe, 2-(4-iodo-2,5-dimethoxyphenyl)-N-(2-methoxybenzyl)ethanamine; 2C-I, 2-(4-iodo-2,5-dimethoxyphenyl)ethanamine; 2C-B, 2-(4-bromo-2,5-dimethoxyphenyl)ethanamine; 25C-NBOMe, 2-(4-chloro-2,5-dimethoxyphenyl)-N-(2-methoxybenzyl)ethanamine; 25H-NBOMe, 2-(2,5-dimethoxyphenyl)-N-(2-methoxybenzyl)ethanamine; 3-MeO-PCP, 1-[1-(3-methoxyphenyl)cyclohexyl]piperidine; 5-IT, 1-(1H-indol-5-yl)propan-2-amine; MXE, methoxetamine; 4-MEC, 4-methylethcathinone; 5-APB, 1-(1-benzofuran-5-yl)propan-2-amine; BZP, 1-benzylpiperazine.

## 4. Discussion

Every year, the European Union Drugs Agency (EUDA) monitors the introduction of new substances on the market with a concern for the health of consumers. Currently, over 950 NPS are under surveillance, with SCRAs being the most reported between 2022 and 2023 [4]. While NPS-related fatalities are identified through post-mortem toxicological analyses, acute intoxications are often under-reported due to misinterpretation of symptoms and lack of toxicological procedures when the subjects are admitted to emergency departments [5]. Indeed, similarly to classic drugs of abuse, NPS consumption may exert a wide range of adverse effects, such as psychopathological ones, as a result of neurotransmitter balance disruption through direct or indirect dysregulation. In particular, SCRAs showed higher affinity for the cannabinoid receptor subtypes (CB1 and CB2), with 2–200 times more potency than that shown by THC [56]. Some SCRAs, such as JWH series or K2, which share their indole structure with serotonin (5-HT) and have an inhibitory property against monoamine oxidase, have been suggested to enhance the activation of 5-HT receptors, leading to serotonin syndrome [57]. A recent study in an animal model revealed that the tyrosine phosphatase PTP1B activation in pyramidal neurons contributes to schizophrenia-like behavior, due to the activation of CB1 receptors, leading to aggressiveness and anxiety [58].

SCs typically have sympathomimetic effects similar to amphetamine, and can act as potent inhibitors of neurotransmitter transporters or releasing agents. The variation in substituents on the α-carbon, or on the amino group and phenyl ring of the cathinone scaffold, lead to different selectivity and/or ligand affinity for dopamine, norepinephrine and 5-HT transporters [59].

Hallucinogens, including natural compounds (psilocybin), phenethylamines (NMBOMe series, 5-IT), arylcyclohexylamines such as 3-methoxy-phencyclidine (3-MeO-PCP), and methoxetamine (MXE) act as partial or full agonists at 5-HT_2A_ receptors with different binding affinities and hallucinogenic effects [60]. The N-benzyl substitution of phenethylamine hallucinogens strongly increases 5-HT_2A_ binding affinity [61].

We investigated evidence reported in the last decade of acute and chronic psychiatric effects on individuals using NPSs. Despite the structural and pharmacological differences in the consumed substances, most individuals displayed the same symptoms, such as alterations in mental status and aggressive behavior involving psychotic symptoms. Other relevant symptoms ranged from paranoia and psychosis to hallucinations and agitation to suicidality. Indeed, suicidality has been frequently linked to SCs and SCRAs [17]. Medium-term effects lasting from one week to eight months were reported in three cases related to SCs and NBOMe nasal insufflation, with occasional visual hallucinations and memory and speech impairments [19,39,40]. Interestingly, only three cases were diagnosed with substance-induced psychotic disorder due to SCRA intake, intravenous injection of MXE and 3-MeO-PCP use [38,54,55]. The three substance-induced psychotic disorder diagnoses were supported by psychometric and neuropsychological rating scales [38], an 18F-fluorodeoxy-glucose positron emission tomography/computerized tomography imaging technique investigating regional brain metabolism, and toxicological analyses [54,55]. These studies highlight the significance of considering multiple factors and utilizing multiple tests (interview, rated scales, diagnostic and screening laboratory tests).

Two HPPD (types I and II), one schizophrenia-like psychosis induced by novel substances and one substance-induced bipolar disorder diagnosis were determined in cases reporting the use of SCRAs, psilocybin, SCs and SCRAs, respectively [21,22,37].

In one of the two HPPD diagnoses, in a patient with declared use of cannabis since the age of 16 years and all types of psychoactive substances, including SCRAs, between 22 and 30 years, the authors reported an initial diagnosis of vegetative dysfunction and myopia peripheral vitreochorioretinal dystrophy after brain MRI and ophthalmologic tests, which resulted in an inconclusive therapy. When the therapeutic program was based on antidepressants, benzodiazepines and anticonvulsants, the patient showed mood improvement, but less improvement in visual disturbances and cenesthopathies, but the follow-up was still ongoing [21].

Beside the high interindividual differences in the reported cases, the present study highlighted that the establishment of a psychiatric diagnosis associated with NPS use is difficult due to several factors. The first limitation is the underestimation and misinterpretation of the symptoms. Indeed, NPS intoxications can lead to different clinical manifestations that are challenging to identify and categorize without the support of further clinical and toxicological investigations. NPSs showed a complex toxicological profile with acute, mid-term and chronic adverse effects. Typically, they are consumed together with other drugs of abuse, enhancing their effects and complicating the accurate association between the symptoms and the specific substance. Moreover, users are often unaware of the substance that they are consuming due to adulteration or counterfeiting of the purchased drug [62]. This misleading information, together with the lack of data on patient clinical history on drug use and/or family history of psychological disorders, can lead to a misdirected initial diagnosis. When toxicological analyses are inconclusive, information on the substances consumed can be obtained only from relatives, friends, police or patient self-reporting. Another consideration is the lack of appropriate toxicological analyses. Routine laboratory screening, such as immunoassays, are not able to identify NPSs: for this reason, further tests with different techniques, such as liquid/gas chromatography coupled to mass spectrometry or high-resolution mass spectrometry, need to be performed [5]. Whenever the toxicology laboratory is not prepared for NPS analyses, the hospital should commit NPS analyses to an external laboratory which is properly equipped. Furthermore, the counseling of national and international agencies prepared for NPSs, such as National Early Warning Systems, the European Drug Agency or the National Institute on Drug Abuse, could be required when NPS intoxication is suspected for further support after diagnosis. Moreover, periodical training on NPS-related issues should be organized for medical staff to prepare them to recognize and manage NPS intoxication cases and ensure the continuous updating of personnel.

Considering the included studies, the general preference for benzodiazepine as the first intervention to manage agitation or aggressive behaviors in clinical practice was confirmed by almost 90% of reported cases, followed by antipsychotics. An effective practice used to manage agitation and prevent face-to face conflicts with healthcare professionals is the program of nonviolent Crisis Intervention^®^ through a verbal de-escalation program [63]. Some authors report that is important for advanced practice nurses to make sure that safety training programs like the Crisis Intervention Program are implemented to avoid the decision to use physical restraint [15]. Others suggest a safe Informed Clinical Management Plan including a mental state assessment through a psychometric rating scale, laboratory analyses with specific methods for the determination of NPSs, medication and physical health monitoring [38]. In many cases patients are discharged due to the resolution of symptoms after 2 to 24 h and further investigations like a follow-up are not performed. Notably, chronic symptoms may have been under-reported due to the lack of a proper follow-up in most cases. Hence, NPS use might have played a role in exacerbating the symptoms of a previous or latent disease or in triggering a new one, especially in cases of past psychiatric symptoms. Regarding this concern, different types of schizophrenia were reported as the main pre-existing psychiatric conditions in NPS consumers with psychiatric symptoms, followed by bipolar disorder. However, psychiatric comorbidities were rarely considered in the NPS intoxication cases due to the lack of proper psychiatric anamnesis and the under-reporting of family history. This consideration underlines the need for the scientific community to examine more thoroughly the clinical outcomes of each individual under the influence of NPSs on admission to hospital and monitor their manifestations over time. Nonetheless, it is undoubtedly challenging to diagnose a mental disorder due to NPS abuse without clear diagnostic criteria and with all the abovementioned difficulties.

It is important to point out that the present review has several limitations. First, our findings cannot be generalized due to the small sample size of the studies. The methodological strategy may be complicated by comorbidities associated with NPS use.
